# Do Mesenchymal Stem Cells Modulate the Milieu of Reconstructed Bladder Wall?

**DOI:** 10.1007/s00005-013-0249-7

**Published:** 2013-08-22

**Authors:** Marta Pokrywczynska, Arkadiusz Jundzill, Magdalena Bodnar, Jan Adamowicz, Jakub Tworkiewicz, Lukasz Szylberg, Robert Debski, Andrzej Marszalek, Tomasz Drewa

**Affiliations:** 1 Department of Tissue Engineering, Ludwik Rydygier Medical College in Bydgoszcz, Nicolaus Copernicus University in Torun, Karlowicza 24, 85-092 Bydgoszcz, Poland; 2 Department of General and Vascular Surgery, Nicolaus Copernicus University in Torun, Bydgoszcz, Poland; 3 Department of Clinical Pathomorphology, Nicolaus Copernicus University in Torun, Ludwik Rydygier Medical College in Bydgoszcz, Bydgoszcz, Poland; 4 Department of Pathology, Poznan University of Medical Sciences, Poznan, Poland; 5 Department of Pediatric Hematology and Oncology, Bydgoszcz, Poland; 6 Department of Urology, Nicolaus Copernicus Hospital, Torun, Poland; 7 Department of Urology, Institute of Oncology, Kielce, Poland

**Keywords:** Bladder regeneration, Cytokines, Matrix metalloproteinases, Mesenchymal stem cells, Tissue engineering

## Abstract

To evaluate the mesenchymal stem cells (MSCs) influence on cytokines and matrix metalloproteinases (MMPs) expression in rat bladder wall regeneration. MSCs cultures from the bone marrow were established. Acellular matrices from the bladder submucosa were prepared. Bladders were reconstructed using cell-seeded (*n* = 5) and unseeded (*n* = 5) grafts. MSCs were injected into the bladder wall (*n* = 5), bladders were incised and MSCs were injected into the circulation (*n* = 5) or were left intact (*n* = 5). Animals were killed after 3 months. Bladder histology and immunohistochemical staining of IL-2, IL-4, IL-6, IL-10, TNF-α, TGF-β1, IFN-γ, MMP-2, and MMP-9 were done. Bladders reconstructed with cell-seeded grafts mimicked native tissue, while unseeded grafts revealed shrinkage and morphological irregularities. There were no morphological changes in bladders of other groups. Different pattern of cytokine and MMP expression was observed. Increased expression of anti-inflammatory cytokines and MMPs in bladder promotes detrusor regeneration.

## Introduction

The gold standard for bladder creation after radical cystectomy is the use of gastrointestinal segments. However, using bowel as a substitute is associated with complications (Nieuwenhuijzen et al. 2008). This encouraged research in tissue engineering for bladder reconstruction. The key idea of this approach is construction of the new bladder wall from autologous cells expanded in vitro and seeded on biodegradable scaffold followed by transplantation for the completion of the regeneration process (Atala et al. 2006; Drewa et al. 2009; Sharma et al. 2011). There are many diseases in which autologous urothelial cells and myocytes cannot be harvested for in vitro bladder wall construction including bladder cancer, which is the most common indication for cystectomy, forms of neuropathic bladder, idiopathic detrusor overactivity, interstitial cystitis or other forms of chronic cystitis (Drewa 2008; Lin et al. 2004; Southgate et al. 2007). Accordingly, there is great need for a new source of cells to construct the bladder wall substitute that would be reliable for clinical applications in the future. Data regarding the molecular aspects of bladder wall reconstruction are sparse, although widespread use of many growth factors to enhance this process was disproven (Kanematsu et al. 2003; Loai et al. 2010). It is known that inflammation hampers regeneration of mammalian tissues (Redd et al. 2004). Mesenchymal stem cells (MSCs) are multipotent stromal cells that can differentiate into muscles. MSCs secrete a variety of bioactive molecules that mediate tissue regeneration and down regulate an inflammatory response (Ding et al. 2011; Yagi et al. 2010). In this regard, MSC-secreted bioactive molecules may have a significant contribution to urinary bladder wall regeneration. The current study was performed to evaluate the MSCs influence on cytokines and matrix metalloproteinases (MMPs) expression in rat bladder wall regeneration.

## Materials and Methods

### Culture and Characterization of MSCs

Femoral bones and urinary bladders were harvested from ten male Wistar rats. Bone marrow was flushed out of the bones with phosphate buffered saline (PBS; PAA, Austria). Cells were cultivated at a density of 5 × 10^5^/cm^2^ at 37 °C and 5 % CO_2_ with complete medium consisting of Dulbecco’s modified Eagle’s medium (DMEM; PAA, Austria) supplemented with 10 % fetal bovine serum (FBS; PAA, Austria), fibroblast growth factor (10 ng/ml; Sigma, Germany), penicillin (100 U/ml; PAA, Austria), and streptomycin (100 μg/ml; PAA, Austria). To confirm the MSCs phenotype, cells were subjected to antigens analysis by flow cytometry. Detached cells from the third passage were washed and resuspended with PBS. Approximately, 1 × 10^6^ cells were incubated with monoclonal primary antibodies conjugated with PE or FITC against CD34 (Santa Cruz Biotechnology, Inc, USA; catalog number sc-7324 PE; 20 μl/sample), CD44 (Millipore, USA; catalog number CBL1508F; 10 μl/sample), CD45 (BD, Pharmingen, USA; catalog number 554877; 0.06 μg/sample) and CD90 (Millipore, USA; catalog number CBL1500F; 10 μl/sample) for 30 min. Expression level of each surface marker was quantified using an EPICS XL flow cytometer (Beckman Coulter, USA). Adipogenic, osteogenic and chondrogenic differentiation was induced as described elsewhere (Le Blanc et al. 2003; Pittenger et al. 1999). Negative control cells were maintained in DMEM/Ham’s F-12 supplemented with 10 % FBS and antibiotics. Adipogenesis was measured by the accumulation of neutral lipids in fat vacuoles, stained with Oil-Red-O. Osteogenesis was confirmed using von Kossa staining. Chondrogenic differentiation was evaluated by Alcian blue staining.

### Grafts

Bladder acellular matrices (BAM) were prepared according to a protocol described by Lai et al. (2003). In brief, the matrices were prepared from rat’s bladders by mechanical removal of epithelial and muscular layers, followed by decellularization in Triton 0.2 % X-100 and 26.5 mmol/L ammonium hydroxide (Sigma, Germany) at 4 °C for 14 days. For detection of MSCs in bladder, the cells were labeled using a PKH-26 red fluorescence cell linker kit (Sigma, Germany), according to the manufacture’s instruction (Lee-MacAry et al. 2001). PKH-26 labeled MSCs from the third passage were seeded on the outer surface of the BAM at a density of 10^6^ cells/cm^2^, incubated to attach for 5 h and cultured for 5 days. Histological analyses of cell-seeded and unseeded BAMs were performed.

### Surgical Procedures

This experiment was approved by the University Ethics Committee (no. 7/2010). Twenty-five syngeneic female Wistar rats weighing between 250 and 300 g were recipients. The animals were randomly divided into five equal groups. Cystoplasty was performed in the first and second group, according to the procedure described previously (Drewa et al. 2009). In brief, rats underwent hemicystectomy, and bladder was augmented with ca. 1 cm^2^ of graft (1 cm × 1 cm × 1.5 mm; length × width × thickness). The anastomosis line was marked by 8.0 monofilament non-absorbable marker sutures to identify the graft borders. In the first and second group, bladders were reconstructed using cell-seeded and unseeded BAM, respectively. In the third group, 10^6^ PKH-26 labeled MSCs were injected into the bladder wall without any additional procedures. In the fourth group, a 1-cm incision of the anterior bladder wall was performed and 10^6^ PKH-26 labeled MSCs were injected into the systemic circulation through the jugular vein. Bladder incision was done to provoke MSCs migration to the injured tissue. The fifth group (control) was left intact. Animals were killed after 3 months. To determine the graft sizes, the distances between un-absorbable marker sutures in filled bladders were measured. Measurements were compared with the initial size of the grafts at surgery. The bladders were harvested for gross and microscopic evaluation.

### Detection of PKH-26 Labeled MSCs

Frozen bladder samples were cut into 8-μm sections and air dried, followed by fixation in 2 % paraformaldehyde for 20 min. After three PBS washes, sections were covered using mounting medium (Dako Cytomation, Denmark). PKH-26 labeled cells were visualized on histological sections under fluorescent microscope (Nicon, Japan).

### Histology

The bladder samples were fixed in 10 % buffered formalin, using routine procedure of tissue processing and embedded in paraffin. Cross-sections of whole bladders were made. The 4 μm thick paraffin sections were stained with hematoxylin and eosin. The connective tissue components and muscle layer were stained according to Masson staining. Urothelial and muscle morphology, capillary density, inflammatory infiltration and nerve regeneration were analyzed and presented as separate values. Since it was impossible to perform classical statistical analyses, the matrix diagrams were used to describe the observed changes and trends. Urothelium was assessed as normal (+) and hyperplastic (++). Smooth muscle layer was evaluated using four point scale corresponding to absent (0), segmental (1), normal with reduced abundance of muscle fibers (2) and normal muscle (3). The intensity of inflammatory infiltration was assessed using four point grading system: lack (0), small focal (1), intensive (2) and lymph follicles formation (3). Capillary density was measured and presented as mean number of vessels <20 μm in diameter per field 500:400 μm. Capillary density scores 0, 1, 2, 3 corresponded respectively to: absent, low (<5 vessels), moderate (5–8 vessels) and high (>8 vessels). Nerves were assessed as present (+) and absent (–). To estimate the amount of muscle fibers, color images of 640 × 480 pixel resolution from each specimen were acquired with a digital camera (Olympus, Japan) running under an imaging analysis program (ImageJ, USA). The muscle tissues were measured for comparison between background and stains. It was quantified by Red–Blue–Green, RBG color histogram, and measure mode. Analysis was repeated for five areas from each specimen.

### Statistical Analysis

Statistical analyses were performed with GraphPad Prism 5.0. Data from each group were evaluated by the Kruskal–Wallis nonparametric one-way analysis of Variance (ANOVA) with *p* < 0.05 considered statistically significant.

### Immunohistochemistry

Immunohistochemical analysis of IL-2, IL-4, IL-6, IL-10, TNF-α, TGF-β1, IFN-γ, MMP-2, and MMP-9 was performed according to the procedure described previously (Marszalek et al. 2011). In brief, tissue sections were incubated with primary antibodies (Table 1). After washing, the sections were overlaid with peroxidase-conjugated anti-mouse, anti-rabbit, or anti-goat secondary antibodies (EnVision or LSAB kit, DAKO, Denmark). Stained samples were analyzed using light microscopy. Five areas of each slide were assessed by two experienced pathologists independently. IL-2, IL-6, IL-10, TNF-α, TGF-β1, IFN-γ, MMP-2, and MMP-9 expressions were evaluated using the immunoreactive score (IRS) according to Remmele and Stegner (1987). The IRS was calculated by multiplying the staining intensity and the percentage of positive cells. The urothelium and stroma were analyzed separately. The staining intensity scores: 0, 1, 2, and 3 correspond to negative, weak, moderate, and strong expression, respectively. The percentage of positive cells scores 0, 1, 2, 3, and 4 correspond to 0, <10 %, 10–50 %, 51–80 %, and >80 %, respectively. It allows a maximum value of 12. Since it was impossible to perform classical statistical analyses, the matrix diagram was constructed to visually determine whether there is a relationship between protein expression and type of intervention. On the basis of IRS, the staining pattern was defined as: negative (IRS 0), weak (IRS 1–4) and strong (IRS 5–12).

**Table 1:** Antibodies used for immunohistochemical staining

Antigen	Distributor/catalog number	Dilution	Incubation	Visualization system
IL-2	R&D/AF-502-NA	2 μg/ml	30 min, 37 °C	LSAB (Dako)
IL-4	Santa Cruz/sc-53084	1:50	16 h, 4 °C	EnVision (Dako)
IL-6	Abcam/ab-6672	1:1200	16 h, 4 °C	EnVision (Dako)
IL-10	R&D/AF-519/NA	5 μg/ml	30 min, 37 °C	LSAB (Dako)
IFN-γ	R&D/AF-585-NA	5 μg/ml	30 min, 37 °C	LSAB (Dako)
TNF-α	Abcam/ab-1793	1:100	16 h, 4 °C	EnVision (Dako)
TGF-β1	Santa Cruz/sc-52893	1:500	16 h, 4 °C	EnVision (Dako)
MMP-2	Santa Cruz/sc-13595	1:50	16 h, 4 °C	EnVision (Dako)
MMP-9	Abcam/ab-58803	1:100	16 h, 4 °C	LSAB (Dako)

## Results

**Fig. 1:**
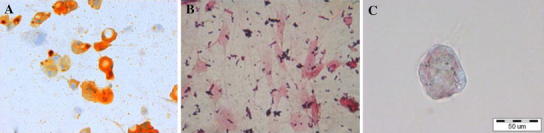
Differentiation potential of MSCs: **a** positive Oil-Red-O staining after adipogenic induction **b** positive von Kossa staining after osteogenic induction and **c** positive alcian blue staining after chondrogenic induction. Light microscope, *scale bar* 50 μm

**Fig. 2:**
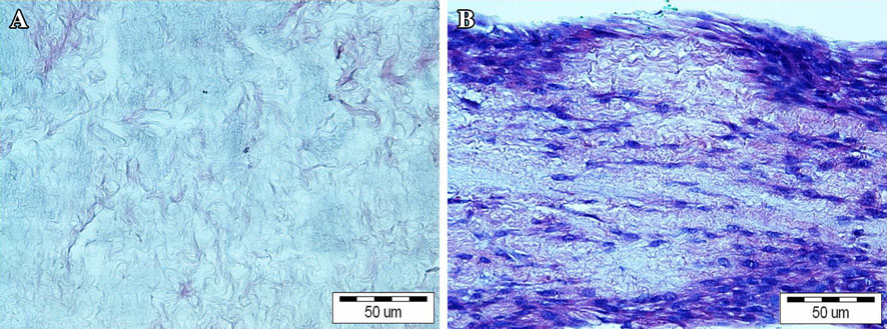
**a** BAM; **b** BAM seeded with MSCs. Hematoxylin and eosine staining, light microscope, *scale bar* 50 μm

Flow cytometry confirmed the homogeneous MSCs phenotype. MSCs derived from third passage were positive for the CD44 (99.5 % of cells) and CD90 (99.7 % of cells) markers and negative for typical endothelial and hematopoietic markers CD34 (0.4 % of cells) and CD45 (0.8 % of cells). MSCs were able to differentiate into adipocytes, osteoblasts and chondrocytes after cultivation in respective media (Fig. 1). Controls showed negative results. No remnants of cell debris were detected throughout the cross-sections of the bladder submucosa after decellularization (Fig. 2a). MSCs seeded on acellular matrices grew in multiple layers. Cell migration through the full depth of the 1.5 mm thick scaffold was observed (Fig. 2b).

**Fig. 3:**
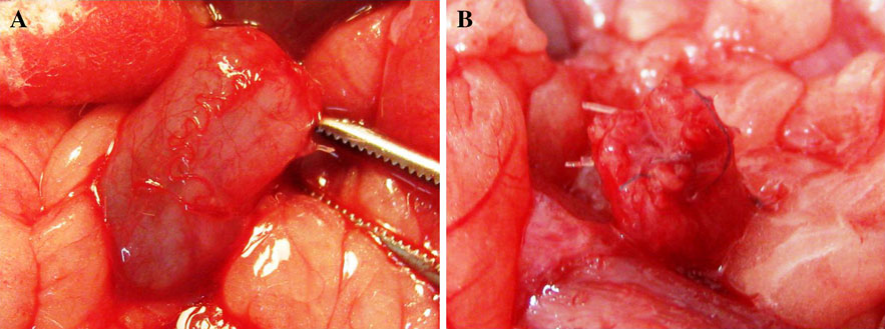
Gross examination of reconstructed bladders. Bladders augmented with cell-seeded **a** and unseeded **b** BAM. Significant graft contracture was observed in bladders reconstructed with unseeded BAM (**b**) while bladders augmented with cell-seeded BAM looked like native bladders (**a**)

**Fig. 4:**
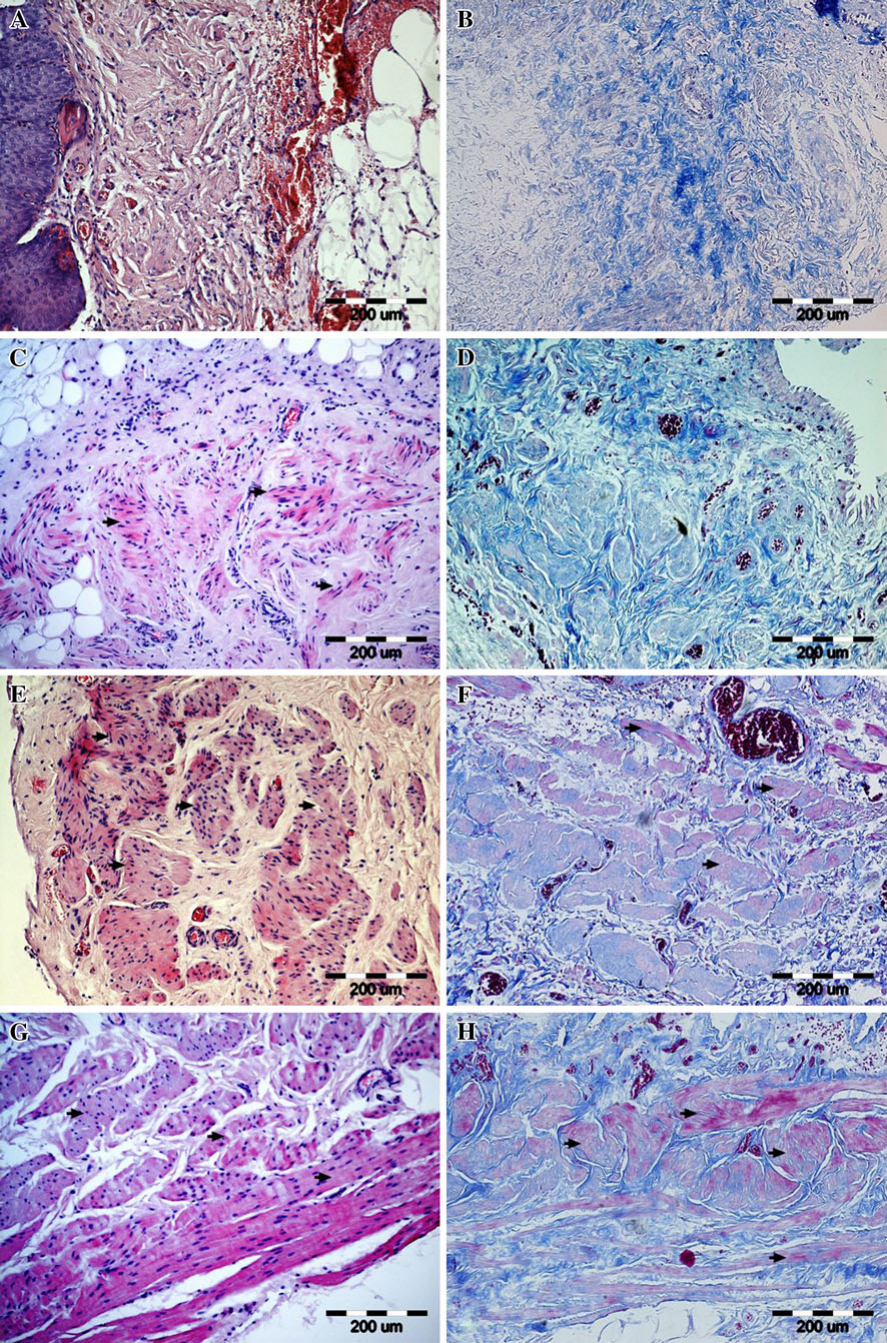
Representative images of the smooth muscle regeneration: (**a**, **b**) absent (0, second group) (**c**, **d**) segmental (1, second group) (**e**, **f**) normal with reduced abundance of muscle fibers (2, first group) (**g**, **h**) normal (3, fifth group-control) in tissue samples stained with hematoxylin and eosine (**a**, **c**, **e**, **g**) and histochemical connective tissue staining method (**b**, **d**, **f**, **h**). Smooth muscles are marked with *arrows*. Light microscope, *scale bar* 100 μm

**Fig. 5:**
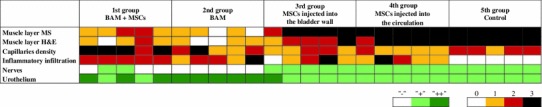
The matrix diagram presenting the histological analysis of bladder samples stained with hematoxylin and eosine (H&E) and Masson staining (MS). Urothelium: normal (+) marked with *light green*, hyperplastic (++) marked with *dark green*. Smooth muscle layer: absent (0) marked with *white*, segmental (1) marked with *yellow*, normal with reduced abundance of muscle fibers (2) marked with *red*, normal muscle (3) marked with *black*. Inflammatory reaction: lack (0) marked with *white*, small focal (1) marked with *yellow*, intensive (2) marked with *red*, lymph follicles formation (3) marked with *black*. Capillary density: absent (0) marked with white, low (1) marked with *yellow*, moderate (2) marked with *red*, high (3) marked with *black*. Nerves: present (+) marked with *green*, absent (−) marked with *white*. *MSCs* mesenchymal stem cells, *BAM* bladder acellular matrix

**Fig. 6:**
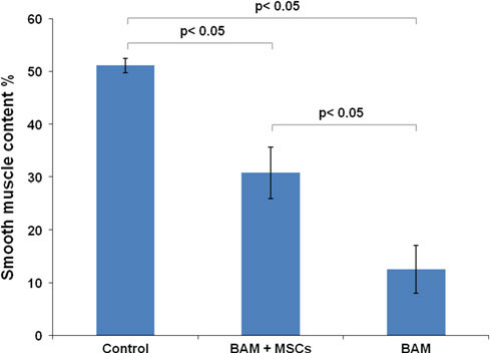
Smooth muscle content in native bladder wall (control group), bladder wall reconstructed using bladder acellular matrix (BAM) seeded with mesenchymal stem cells (MSCs) (first group) and unseeded BAM (second group), respectively. Differences between the control and first group, first and second group as well as between the control and second group were statistically significant *p* < 0.05. Values are expressed as mean (SD)

**Fig. 7:**
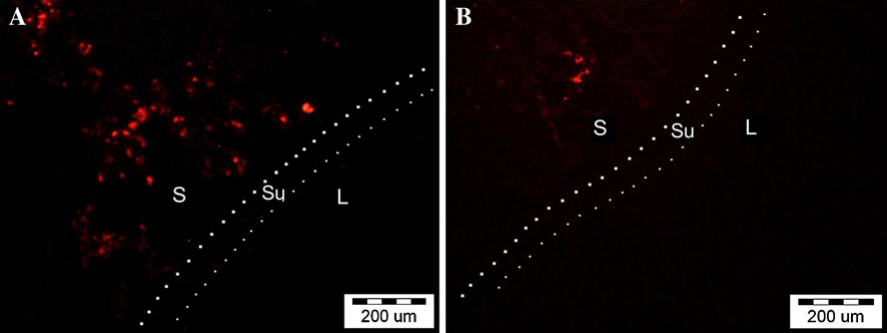
PKH-26 labeled cells: **a** in reconstructed bladder wall (first group) **b** injected to the circulation and migrated to the injured bladder (fourth group). *S* stroma, *Su* submucosa, *L* bladder lumen. Fluorescence microscope, *scale bar* 200 μm

All the animals survived the observation period. No urinary leakage or calcifications were observed. Reconstructed tissue in the first group was similar to the native bladder wall on gross examination (Fig. 3a). Graft shrinkage (54 ± 11 %, mean ± SD) in the second group was observed (Fig. 3b). The histological examination detected the presence of three bladder layers in the first, third, fourth, and fifth groups. Evaluation of structure of muscular layer revealed a normal muscle in the third, fourth and control groups. Muscle layers in the apical parts of reconstructed bladders were absent (Figs. 4a, b; 5) or extremely thin when augmented with acellular matrices (Figs. 4c, d; 5). The detrusor fibers content was significantly higher in bladders reconstructed with cell-seeded matrices (Figs. 4e, f; 5). Digital image analysis showed that bladders reconstructed with cell-seeded matrices did not achieve the same percentage of muscle fibers as the native bladder, but they were statistically more abundant in detrusor muscle when compared to bladders reconstructed with acellular matrices (Fig. 6). However, the quantity and organization of muscle fibers were irregular when compared to native tissue (Fig. 4e, f, g, h). Evidence of neovascularization was seen on the surface of both seeded and unseeded implants, but capillary density was the highest in bladders augmented with cell-seeded grafts (Fig. 5). According to presence or lack of nerves as well as presence or lack of epithelial hyperplasia, there was well visible dichotomic separation of control, third and fourth groups versus first and second groups. In the former there was lack of urothelium hyperplasia, but nerves were present. While in the latter the opposite was observed, namely there was urothelial hyperplasia and almost in all cases lack of nerves. Nerve regeneration was observed in two bladders reconstructed with cell-seeded grafts, but not in bladders augmented with acellular matrices (Fig. 5). An elevated mononuclear cell infiltration was observed in all experimental groups (Fig. 4). Fluoresce analysis confirmed the presence of implanted cells in bladders 3 months after surgery. The numerous PKH-26 labeled cells were detected in augmented bladders. These cells account for 20 % of all cells repopulating reconstructed bladder wall (Fig. 7a). Only single PKH-labeled cells were observed in fourth group, where a 1-cm incision of the anterior bladder wall was performed and MSCs were injected into the systemic circulation (Fig. 7b). Numerous cells migrated to another tissues and organs, especially, spleen, liver and bone marrow.

**Fig. 8:**
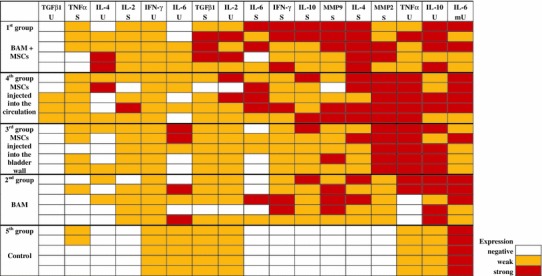
The matrix diagram presenting the cytokines and MMP expression ranked from the weakest to the strongest. Immunoreactive score (IRS): negative (IRS: 0) marked with *white*, weak (IRS: 1–4) marked with *yellow*, and strong (IRS: 5–12) marked with *red*. *BAM* bladder acellular matrix, *MSCs* mesenchymal stem cells, *U* urothelium, *mU* cell membrane of urothelium, *S* stroma

**Fig. 9:**
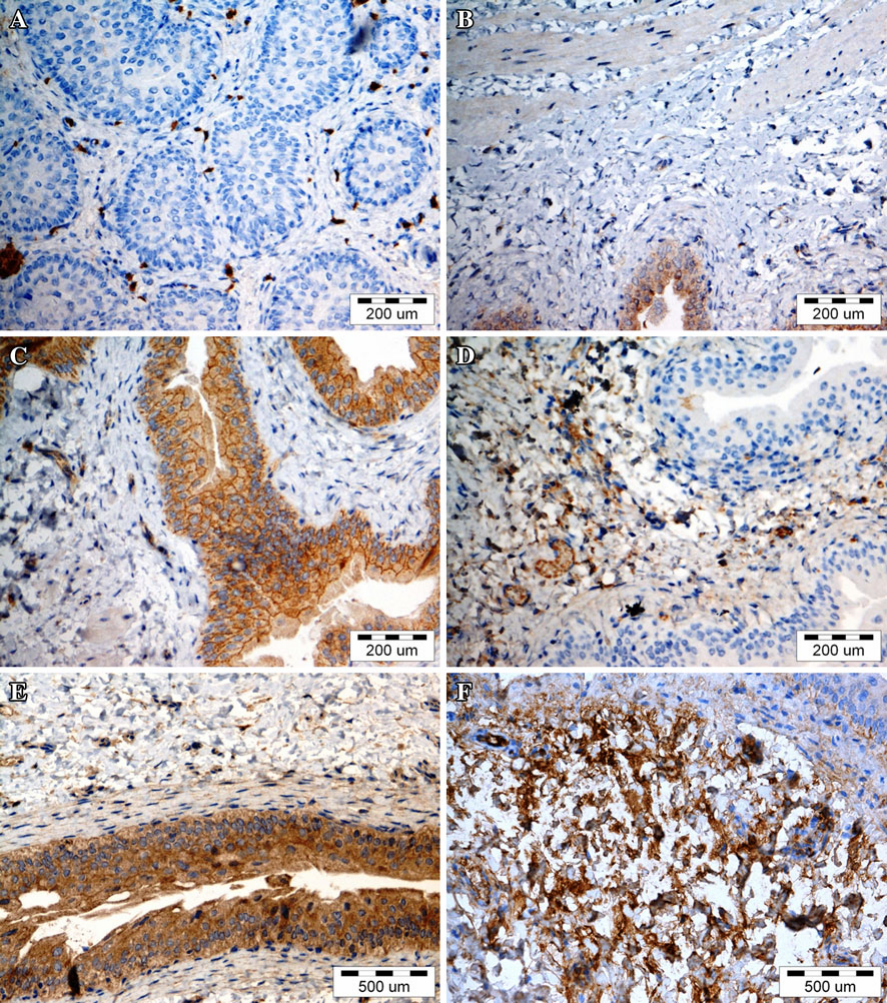
Representative images of cytokines and matrix metalloproteinases expression. **a** negative expression of TGF-β1 in urothelium (first group) **b** negative expression of TNF-α in stroma (second group) **c** weak cytoplasmic and strong membrane expression of IL-6 in urothelium (fourth group) **d** weak expression of IL-4 in stroma (third group) **e** strong expression of IL-10 in urothelium (third group) **f** strong expression of MMP-9 in stroma (first group). Immunohistochemical staining, light microscope, *scale bar* 200 and 500 μm

The profile of cytokine and MMP expression in bladders changed depending on the type of treatment (Fig. 8). Cytokine expression was mainly observed in the cytoplasm with the exception of IL-6, which indicated a mixed cytoplasmic and membranic expression (Fig. 9c). The expression pattern was considerably changed in the first and fourth groups. IL-4, IL-10, IFN-γ, MMP-2, and MMP-9 were elevated in the bladder stroma of the experimental groups. An interesting finding is weak cytoplasmic expression of IL-2, IL-6, IL-10, TNF-α and IFN-γ in urothelium in the control group. The third and fourth groups represent strong expression of TNF-α in urothelium coexisting with strong expression of MMP-2 in bladder stroma (Fig. 8). Representative photographs of immunohistochemical staining, presenting negative, weak and strong expression for selected cytokines and MMPs are shown in Fig. 9.

## Discussion

One of the new trends in tissue engineering is scaffolds integrated with growth factors (“smart matrices”). Although it has been demonstrated that these smart matrices promote urinary tract regeneration, it should be strongly emphasized that a non-physiological concentration or improper selection of growth factors can lead to tissue overgrowth, fibrosis, or other complications (Kanematsu et al. 2003; Loai et al. 2010; Nuininga et al. 2010). It has been suggested that alternative sources of autologous cells for bladder detrusor regeneration in cancer patients could be bone marrow, fat tissue, or skin/hair follicles (Drewa 2008; Drewa et al. 2009; Shukla et al. 2008; Zhu et al. 2010). All these data are focused on regeneration effects, but no information describing the molecular basis of this process can be found in literature. Understanding that molecular aspects of bladder regeneration are fundamental for future research in this field, we investigated the efficacy of bone marrow MSCs in improving the bladder muscle regeneration and analyzed the cytokines and MMPs expression in this process.

There was no need to use cell-enhancing regeneration of the urothelium because of its high potential for physiological self-renewal. Three months after the reconstruction, the urothelial covering was complete. The hyperplasia of the urothelium that was observed in bladders reconstructed with unseeded grafts could be an alarming sign of urothelial dysfunction and improper urothelial regeneration engendered by inflammation. At 3 months postoperatively, there were no remains of BAM. Applying acellular matrix to bladder wall reconstruction yielded only partial regeneration of the muscle layer. Our study confirmed that the use of MSC-seeded matrix is a crucial requirement to achieve muscle layer and a normal structure of bladder wall. We have found that implanted MSCs accounted pretty good percentage of all cells repopulating reconstructed bladder wall. The number of cells detected in reconstructed bladder wall accounted for about 30 % of total number of transplanted cells. The smooth muscle ontogeny in reconstructed bladder wall has not been defined. We think that transplanted bone marrow derived cells differentiated into smooth muscle cells on acellular matrix grafts in response to the environment created by smooth muscle cells. Sharma indicated that more than 90 % of MSCs used for reconstruction of urinary bladder differentiated into the smooth muscle cells (Sharma et al. 2011). Shukla showed that only 2–3 % of bladder smooth muscle cells were derived from transplanted stem cells (Shukla et al. 2008). Smooth muscle regeneration is probably the result of several overlapping processes not only differentiation of transplanted MSCs but also migration of smooth muscle cells or their progenitors from native bladder wall or even stem cells from circulation (Kanematsu et al. 2005; Sharma et al. 2011; Shukla et al. 2008; Wu et al. 1999). High PKH-26 expression in reconstructed bladders is probably connected with low proliferation rate of differentiated cells. A number of in vivo studies have shown that systemically infused MSCs could migrate to injured tissues and exert therapeutic effects (Chapel et al. 2003; Chavakis et al. 2008). We indicated that MSCs injected to the systemic circulation migrate to the injured bladder tissue.

Regeneration of bladder tissue is a challenge because, in the adult mammals, most wounds heal by repair, which leads to scar formation. Independent observations of adult healing following injury have shown that in the majority of organs, excised epithelial tissues and basement membranes regenerate spontaneously following excision while some elements of stroma does not. Stromal regeneration in adult mammals can be induced, but requires tissue-engineering techniques, which was confirmed by our study. In contrast to human adults, the mammalian fetus and amphibians, heals wounds spontaneously by regeneration (Menger et al. 2010; Yannas 2005). This regeneration is a sequential cascade of overlapping processes resulting in functional tissue formation. It can be speculated that regeneration replicates organogenesis (Yannas 2005). The cytokines and MMPs play a crucial role in this process. It is well known that early fetal mammalian as well as amphibian wounds exhibit very little, if any, inflammatory response during regeneration (Menger et al. 2010; Redd et al. 2004; Yannas 2005). The cytokines are generally divided into “pro-inflammatory” (IL-2, IL-6, IFN-γ, and TNF-α) and “anti-inflammatory” (IL-4, IL-10, and TGF-β) as determined by their range of actions, although many cytokines exert mixed pro- and anti-inflammatory effects (Abbas and Lichtman 2003). MMPs degrade extracellular proteins and thus play an essential role in tissue remodeling (Visse and Nagase 2003). The absence of inflammation may be at least in part responsible for the rapid and scarless wound healing (Redd et al. 2004). We postulate that MSCs activated within the environment of the injured bladder upregulate anti-inflammatory cytokines enhancing tissue regeneration. In this study, the cytokines and MMPs expressions were evaluated over a long period of 3 months. This is very important period of tissue healing, determining the quality of reconstructed tissue, not only a morphological structure but also its function (strength, elasticity and flexibility). We think that only evaluation of reconstructed bladder wall after long-term observation can lead to relevant conclusions. IL-2, IL-4, IL-6, IL-10, TNF-α, TGF-β1, IFN-γ, MMP-2, and MMP-9 were evaluated because they are involved in the process of tissue repair and regeneration, moreover, TGF-β1, IL-6, and MMPs are secreted by MSCs (Burdon et al. 2011). Urothelium and bladder stroma stimulated different cytokine expression profiles depending on type of intervention. These results suggest that urothelium and stroma were affected differently by MSCs. The expression of cytokines in the native bladder was observed mainly in urothelium. Our data demonstrated that any interventions reversed this profile. This phenomenon was the best marked in the MSCs-treated groups. On the other hand, expression of IL-10 in urothelium and MMP-9 in stroma was strong in reconstructed bladders regardless of whether MSCs were transplanted or not. However, expressions of IL-4, TGF-β1, and IFN-γ were higher in the stroma of bladders reconstructed with cell-seeded BAM compared to bladders grafted with acellular matrix. All of these cytokines regulate the extracellular matrix remodeling; moreover, IL-4 and TGF-β1 depress the immunological response. IL-4 and TGF-β1 stimulate and IFN-γ inhibits extracellular matrix protein synthesis (Chen et al. 2005). The most obvious difference between the first and second group concerns the expression of TGF-β1 and IL-4. TGF-β1 and IL-4 are anti-inflammatory cytokines with a wide range of biological activities. In many pathologies, the excessive or prolonged expression of these cytokines contributes to tissue fibrosis (Weedon 2002). In this study, we observed no association between the increased expression of TGF-β1 or IL-4 and fibrosis in gross and histological examinations. It has been shown that TGF-β1 modulates cell growth and differentiation of both urothelium and bladder smooth muscle (de Boer et al. 1994; Kurpinski et al. 2010). TGF-β1 stimulates differentiation of MSCs into smooth muscle cells in vitro (Kurpinski et al. 2010). It is quite likely that TGF-β1 and IL-4 play an important role in bladder regeneration and regulate proper bladder wall remodeling following injury. Our study also indicated that strong expression of TGF-β1 coexists with increased angiogenesis, which is an important factor influencing graft survival (Ferrari et al. 2009). This finding indicates that exogenous TGF-β1 and IL-4 could be used potentially for construction of smart biomaterials to enhance bladder wall regeneration as cytokines with anti-inflammatory properties. The pattern of cytokines and MMPs expression in bladders was comparable regardless of whether the cells were injected locally (third group) or systematically (fourth group).

Based on the results of this study, we can speculate that there is some association between type of secreted MMP and extent of surgical intervention. MMP-2 was secreted in bladders that underwent less invasive surgery (the third and fourth groups) while MMP-9 expression appeared mainly in bladders reconstructed after hemicystectomy. These findings show that MMP-2 and MMP-9 play different roles in bladder healing. It is quite likely that MMP-9 facilitates smooth muscle migration. We noticed that TNF-α expression in urothelium coexisted with MMP-2 expression in bladder stroma. This observation has been confirmed by others (Han et al. 2001). The reason for the increased level of TNF-α in the urothelium of the third and fourth groups is unknown and requires future investigation.

The process of tissue remodeling following biomaterial implantation is associated with a robust macrophage response beginning as early as 2 days post implantation and continuing for several months (Brown et al. 2012). Macrophages have been classified into two major types: M1 (classically activated; pro-inflammatory) and M2 (alternatively activated; regulatory, homeostatic). M1 and M2 macrophages play distinct roles in tissue remodeling. M1 response with elevated expression of TNF-α, IL1 and IL6 is usually observed in early phases of healing, whereas M2 response with high level of IL-10 and TGF-β in later phases (Hao et al. 2012). In addition, the IL-10 expressed by M2 macrophages can promote the production of IL-4 by Th2 cells (Mantovani et al. 2009). On the other hand, IL-4 stimulates M2 macrophages phenotype (Lee et al. 2011). In this study, the macrophage phenotype has not been evaluated; however, on basis of cytokine pattern we can speculate that in bladders augmented with cells seeded grafts (high expression of IL-4 and TGF-β) it would be M2 macrophages. We believe that the increased expression of anti-inflammatory cytokines and MMPs in the bladder stroma triggered the regeneration of the muscle layer, which is the most important part for successful urinary bladder regeneration. These results strengthen the possibility for the successful clinical application of MSCs in bladder regeneration in the future.

The main weakness of this study is lack of appropriate control for the group 4 (bladder wall incision together with MSCs injection into the blood circulation). We used an untreated animal as a control for the group 4, however, it should be emphasized that the best control for this group would be bladder wall incision group. Moreover, although 1 × 10^6^ MSCs were seeded on each scaffold, it is unknown exactly how many cells adhered to the scaffold, but finally the cell number was similar in each group.

In conclusion, the results of this study suggest the role of anti-inflammatory cytokines and MMPs in urinary bladder smooth muscle regeneration. These findings may improve the understanding of the role of MSCs in the bladder wall regeneration process.
